# Severity Matters: How COVID-19 Severity Impacts Long-Term Effects on Symptoms, Physical Activity and Functionality—An Observational Study

**DOI:** 10.3390/healthcare13030333

**Published:** 2025-02-06

**Authors:** Laura Pérez-Gisbert, Concepción Morales-García, José Antonio Sánchez-Martínez, María Victoria González-Gutiérrez, Marie Carmen Valenza, Irene Torres-Sánchez

**Affiliations:** 1 Physical Therapy Department, Faculty of Health Sciences, University of Granada, Avenida de la Ilustración nº 60, 18016 Granada, Spain; laurapg98@correo.ugr.es (L.P.-G.); cvalenza@ugr.es (M.C.V.); 2 Pneumology Service, Virgen de las Nieves University Hospital, Avenida de las Fuerzas Armadas nº 2, 18014 Granada, Spain; concepcion.morales.sspa@juntadeandalucia.es (C.M.-G.); josea.sanchez.martinez.sspa@juntadeandalucia.es (J.A.S.-M.); mvglezgut007@gmail.com (M.V.G.-G.)

**Keywords:** COVID-19, symptoms, physical activity, functionality

## Abstract

Background/Objectives: The existing literature has described the common symptoms and long-term effects of coronavirus disease (COVID-19). However, there is a lack of detailed information on how different degrees of disease severity affect survivors differently. This study aims to fill that gap by evaluating the symptoms, physical activity, and functionality of COVID-19 survivors across a spectrum of severity levels, comparing them with those of healthy individuals. Methods: An observational study was carried out following the Strengthening the Reporting of Observational Studies in Epidemiology (STROBE) criteria and checklist. Participants were divided into 5 groups based on COVID-19 severity according to the World Health Organization classification: healthy (COVID-19-negative), mild (symptomatic without pneumonia or dyspnoea), moderate (pneumonia and dyspnoea without hospitalisation), severe (severe pneumonia requiring hospitalisation), and critical (severe pneumonia with admission to the intensive care unit). Descriptive variables, symptoms (Fatigue Borg Scale, Fatigue Impact Scale, Fatigue Severity Scale, Dyspnoea Borg Scale, Visual Analogue Scale, Hospital Anxiety and Depression Scale, and European Quality of Life-5 Dimensions), physical activity (the International Physical Activity Questionnaire) and functionality (Patient-Specific Functional Scale, Short Physical Performance Battery, Arm Curl test, and 2 min step test) were measured. Results: A total of 304 participants were included: healthy (*n* = 42), mild (*n* = 143), moderate (*n* = 49), severe (*n* = 52), and critical (*n* = 18) COVID-19 patients. The impact of COVID-19 on surviving patients varies significantly with the severity of the disease. The results show that the hospitalisation time, age, and comorbidities of the patients are greater in those with a greater severity of the disease. Patients with more severe COVID-19 also experience greater frailty, dysphagia, fatigue, dyspnoea, and pain. Additionally, those with severe cases have poorer overall health, reduced physical activity, and diminished functionality. No evidence of post-COVID-19 anxiety or depression is found in the sample, even considering the timeframe between the negative test and the assessment. Conclusions: Patients with higher COVID-19 severity (severe or critical) experience more symptoms than those with lower COVID-19 severity (mild or moderate). Additionally, those with severe cases have poorer overall health, reduced physical activity and diminished functionality. Register: Clinicaltrials.gov: NCT05731817.

## 1. Introduction

The severe acute respiratory syndrome coronavirus 2 (SARS-CoV-2) causes coronavirus disease (COVID-19). The severity of COVID-19 can depend on extrinsic factors such as demographic data, the quality of healthcare or vaccines, or intrinsic factors such as susceptibility [1]. Depending on the severity, symptomatic COVID-19 patients can be classified into mild, moderate, severe, and critical. There is also the possibility of being asymptomatic [2]. In these cases, cytokines and inflammation markers are at the same levels as those of healthy people; that is, they do not generate a detectable inflammatory response [3]. In symptomatic cases, 40% are mild, 40% moderate, 15% severe, and 5% critical [2].

A mild disease is considered when patients are symptomatic but they do not present viral pneumonia or dyspnoea [2]. The disease is moderate when patients have pneumonia but without severe signs. Mild and moderate forms do not require hospitalisation. In severe disease, patients present severe pneumonia and require hospital admission. Finally, the disease is critical when the patient require admission to the intensive care unit (ICU) [4].

Regardless of the severity of disease, common symptoms are fever, dry cough, fatigue, expectoration, dyspnoea, pain sore throat, headache, myalgia or arthralgia, chills, nausea and/or vomiting, nasal congestion, diarrhoea, haemoptysis, and conjunctival congestion [5]. Moreover, in hospitalised patients, the most frequent symptoms are fever, fatigue, and cough [6].

When symptoms persist beyond 12 weeks and they are not related to an alternative diagnosis, it is considered post-COVID-19 syndrome (PCS). Over 30% of survivors, and up to 80% of hospitalised patients, experience persistent symptoms [7]. The pathophysiological basis of PCS is unknown, although there are three possible theories: the persistence of the virus in reservoirs, damage caused by autoimmunity or an excessively deregulated immune response. Whatever the cause of these persistent symptoms, multiple organs may be involved. The main persistent symptoms are fatigue, dyspnoea, chest pain, headache, the loss of taste or smell, muscle and joint pain, depression/anxiety, insomnia, body itching, palpitations, tachycardia, anorexia, tingling in the fingertips of fingers, and mental confusion [8].

While the existing literature outlines the common symptoms and long-term effects of COVID-19 [7,8,9], it lacks detailed insights into how varying degrees of disease severity impact survivors differently. Some studies suggest that the long-term effects of COVID-19 occur more frequently in hospitalised (severe or critical) patients [10,11,12]. However, there is evidence that these persistent symptoms can also affect patients with mild or moderate cases [7]. This highlights the importance of investigating all degrees of disease severity. Lopez-Leon et al. [9] in their systematic review and meta-analysis analysed more than 50 documented long-term effects in post-COVID-19 patients and estimated the prevalence of each persistent symptom. Their findings revealed that fatigue (58%), headache (44%) and attention disorders (27%) were among the most common persistent symptoms. However, the authors stressed the crucial need for future studies to stratify the results according to the severity of COVID-19 during the acute phase in order to better understand how disease progression influences long-term effects.

Based on these observations, our study seeks to directly address this gap through a comprehensive and comparative analysis of symptoms, physical activity, and functionality as a function of the initial disease severity. By comparing COVID-19 survivors with different levels of severity with healthy individuals, this research study aims to provide valuable information on the relationship between disease severity and its long-term impacts, contributing to the optimisation of rehabilitation strategies.

The aim of this study is to evaluate the symptoms, physical activity, and functionality of COVID-19 survivors across a spectrum of severity levels and compare them with those of healthy individuals.

## 2. Materials and Methods

### 2.1. Design

An observational study was carried out. The Strengthening the Reporting of Observational Studies in Epidemiology (STROBE) criteria and checklist were applied (see Appendix A).

### 2.2. Ethical Aspects

The study protocol was approved by the ethics committee of the Biomedical Research Ethics Committee of Andalucía. It was conducted in accordance with the 1975 Helsinki Declaration, revised in 1983. Moreover, all participants provided written informed consent prior to their inclusion in the study.

### 2.3. Participants

Hospitalised patients were recruited from Virgen de las Nieves University Hospital (Granada, Spain) after being discharged from the hospital. Healthy and non-hospitalised patients were recruited in Granada, Spain, through snowball sampling where initial participants referred other eligible candidates. A total of 304 participants were evaluated between February 2022 and June 2023.

Participants were divided into 5 groups based on COVID-19 severity according to the World Health Organization (WHO) classification [2]: healthy (*n* = 42), mild (*n* = 143), moderate (*n* = 49), severe (*n* = 52), and critical (*n* = 18).

Common criteria for all participants: being adults of legal age; having basic knowledge and access to internet; willingness to participate in the study; and acceptance of informed consent.

Inclusion criteria for groups include healthy: never tested positive for COVID-19 according to their medical history and self-reports; mild: tested positive for COVID-19, symptomatic without viral pneumonia, hypoxia or dyspnoea and non-hospitalised; moderate: tested positive for COVID-19, symptomatic with pneumonia and dyspnoea and non-hospitalised; severe: tested positive for COVID-19, symptomatic with severe pneumonia and hospitalised; and critical: tested positive for COVID-19, symptomatic with severe pneumonia and admission to the ICU.

Exclusion criteria for all participants: severe comorbidities interfering with the ability to perform the study; and mental, physical or organic problems that, under medical criteria, could pose a risk to the subject.

### 2.4. Data Collection

A telephone call was made to explain the study and schedule their evaluation. On the day of the appointment, a link to a video call was provided, during which data collection was conducted. For hospitalised patients, evaluations were performed after discharge and at least 1 week after a negative test result. Similarly, non-hospitalised patients were assessed at least 1 week after receiving a negative test result. In order to minimise the impact of recall bias, the questions were carefully designed to focus on concrete and specific events, avoiding interpretations and generalisations. In addition, “double-check” questions were used, where possible, to identify possible inconsistencies [13].

The collected data were stored in an Excel spreadsheet, which adhered to necessary security measures to protect confidentiality.

### 2.5. Study Variables: Measuring Tools

#### 2.5.1. Study Variables: Descriptive Characteristics

Age, gender, BMI, days between the negative test and assessment date, days of hospitalisation, and days in ICU: online questions.

Comorbidities: The Charlson Comorbidity Index. No comorbidities are considered 0–1 point, low comorbidities 2 points and high comorbidities ≥ 3 points [14].

Frailty: The Clinical Frailty Scale (CFS) assesses the functional status of patients. The score ranges from 0 (best functional status) to 9 points (terminal disease). The FRAIL frailty scale (FRAIL) categorises patients as frail (3–5), pre-frail (1–2), or robust (0) [15].

Dysphagia: The Eating Assessment Tool (EAT-10) is a 10-item questionnaire with scores ranging from 0 (no problem) to 4 (serious problem). A total score of ≥3 indicates potential swallowing issues [16].

#### 2.5.2. Study Variables: Symptoms

Fatigue: The Borg Scale is a subjective scale ranging from 0 (no fatigue) to 10 (extreme fatigue) [17]. To assess the impact of fatigue, the Fatigue Impact Scale (FIS) was used. It is a questionnaire composed of 21 items. Each response is evaluated with a score from 0 (never) to 4 (almost always). Higher scores indicate a greater impact of fatigue. Thirty-eight is the cut-off point for fatigue [18]. The Fatigue Severity Scale (FSS) evaluates the severity of fatigue. It is a 9-item self-report questionnaire rated on a 7-point Likert scale. The total score is a maximum of 63 points. The cut-off point to determine the presence of fatigue is 36 [19].

Dyspnoea: The Borg Scale is a subjective scale that relates the perceived respiratory difficulty from 0 (no dyspnoea) to 10 (extreme dyspnoea).

Pain: The Visual Analogue Scale (VAS) is a subjective rating scale that ranges from 0 (the absence of pain) to 10 (maximum pain).

Anxiety and depression: The Hospital Anxiety and Depression Scale (HADS) is made up of 14 items divided into anxiety and depression. The maximum score ranges between 0 and 42 points. A higher score indicates greater anxiety and depression. Thirteen is the cut-off point to determine the presence of anxiety and depression [20].

Quality of life: European Quality of Life-5 Dimensions (EuroQol-5D) is divided into 2 sections. The first part assesses mobility, personal care, daily activities, pain/discomfort, and anxiety/depression with severity levels coded as 1 (no problems), 2 (some problems), and 3 (many problems). The second part is a VAS where participants rate their current health state from 0 (worst) to 100 (best) [21].

#### 2.5.3. Study Variables: Physical Activity and Functionality

Physical activity: The International Physical Activity Questionnaire (IPAQ) consists of 7 questions about physical activity carried out in the 7 days immediately prior to the assessment. Subjects can be divided into 3 categories: low or inactive (category 1), moderate (category 2) or high (category 3) [22].

Functionality: The Patient-Specific Functional Scale (PSFS) is a questionnaire in which patients name 3 activities that they find difficult or unable to perform due to COVID-19, rating each from 0 (disability) to 10 points (perfect ability) [23]. Physical test: Short Physical Performance Battery (SPPB) evaluates balance, walking speed and lower limb strength. A score < 10 points indicates frailty and an increased risk of disability and falls; the Arm Curl test measures the number of push-ups performed in 30 s using a 1 kg weight; and the 2 min step test (2MST) is a test in which participants walk in place, taking as many steps as possible in 2 min.

### 2.6. Statistical Analysis

All statistical analyses were performed using IBM SPSS version 28.0 (IBM, Armonk, NY, USA). Normally distributed numerical data were compared using one-way analysis of variance (ANOVA) and post hoc Bonferroni tests and were expressed as means ± standard deviation. Categorical variables were compared using chi-square tests and presented as numbers and percentages. A *p*-value of <0.05 indicated a significant difference. There were no missing data.

## 3. Results

**Figure 1:**
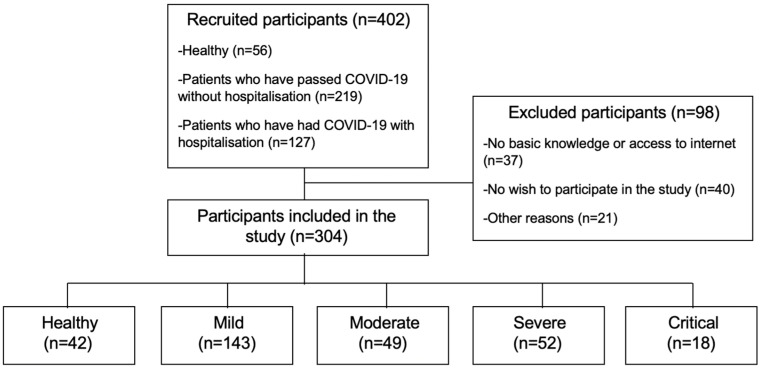
Flow diagram.

A total of 304 participants were divided into five groups: healthy (*n* = 42), mild (*n* = 143), moderate (*n* = 49), severe (*n* = 52), and critical (*n* = 18) COVID-19 patients (see Figure 1).

### 3.1. Results: Descriptive Characteristics

Table 1 shows that age increases as the severity of COVID-19 increases, with significant differences between groups. Participants of both genders were included.

Days of hospitalisation were longer for critical patients compared to severe cases.

The Charlson Comorbidity Index showed “high comorbidities” for critical patients, “mild comorbidities” for severe, moderate, and mild patients, and “no comorbidities” for the healthy group.

The CFS showed that frailty increased as the severity of the disease increased, with significant differences between groups. FRAIL added that healthy, mild and moderate groups were “robust” compared to severe and critical groups who were “pre-frail”, with significant differences.

Finally, the perception of dysphagia was greater the more severely the patients experienced COVID-19. However, only the critical group exceeded the cut-off point (≥3), presenting dysphagia with significant differences.

### 3.2. Results: Symptoms

The symptoms are shown in Table 2. For fatigue, the Borg scale showed that fatigue measured at rest and during light, moderate, and intense exercise increased along with the severity of COVID-19. Except at rest, this was higher for the moderate group. Differences were significant in several groups. The results of the FIS showed that the moderate, severe, and critical groups of patients presented fatigue, unlike the healthy and mild groups, who did not pass the cut-off point. Differences were significant between some groups. This coincides with the FSS, which also indicated that the moderate, severe, and critical groups presented fatigue when passing the cut-off point, unlike the healthy and mild groups. Furthermore, among those with fatigue, the severity of fatigue was greater as the severity of COVID-19 increased, with significant differences.

Dyspnoea increased with the increasing severity of COVID-19. Significant differences were found between groups.

The pain results measured with the VAS were 1.33 ± 2.21 in the group with less pain and 3.67 ± 3.56 in the group with more pain. It increased as the severity of COVID-19 increased with significant differences.

According to the results of the HADS, no group exceeded the cut-off point (>13) to determine the presence of anxiety and/or depression. However, it was observed that the more severe cases presented higher scores in anxiety and depression compared to the milder cases.

EuroQol-5D for quality of life showed that patients in the different groups did not have relevant problems in any of the subscales measured. However, the VAS showed a worsening of health status as the severity of COVID-19 increased, with significant differences.

### 3.3. Results: Physical Activity and Functionality

The results of physical activity and functionality are shown in Table 3. The IPAQ showed a higher weekly metabolic expenditure and therefore a higher level of physical activity in healthy patients, followed by mild, severe, moderate, and critical patients. No significant differences were found between groups.

For functionality, the PSFS showed that the functional limitation was greater in patients who had had COVID-19 in a more severe form, with significant differences. The results of the SPPB showed that all participants who had had COVID-19, unlike healthy ones, exceeded the cut-off point, so they were frail and at a greater risk of disability and falls. Furthermore, functionality worsened as the severity of COVID-19 increased. Significant differences were found in several groups. The Arm Curl test showed a lower number of repetitions, with both arms, in those patients who had had COVID-19 in a more severe form. Differences were significant in some groups. Finally, the 2MST also showed a greater number of steps in healthy patients, followed by mild, moderate, severe, and critical patients. Furthermore, significant differences were found between groups.

## 4. Discussion

The objective of this study was to fill a gap by evaluating the symptoms, physical activity, and functionality of COVID-19 survivors across a spectrum of severity levels, comparing them with those of healthy individuals. The results show that the length of hospitalisation and the age of the patients are greater in patients with a greater severity of the disease. Moreover, as the severity of COVID-19 increases, there are also more comorbidities and greater frailty, the perception of dysphagia, fatigue, dyspnoea, and pain. Finally, the greater the severity of COVID-19, the lower the state of health, physical activity, and functionality. Although none of the groups exceed the established cut-off point for determining the presence of post-COVID19 anxiety and/or depression, higher anxiety and depression scores are observed in cases with higher severity compared to those with lower severity.

### 4.1. Discussion: Descriptive Characteristics

The average age of participants increased with the severity of COVID-19, consistent with Mansell et al. [24]. They reported that older patients tend to experience more severe COVID-19 compared to younger individuals. Both genders were included in each group. However, a tendency can be seen regarding an increase in the male gender of the most severe forms compared to the mildest. This aligns with the existing literature [25] suggesting that men are at a higher risk of severe COVID-19 outcomes, although further research is needed. Additionally, we found that patients in a critical condition had longer hospitalisation times compared to those with severe cases, which corroborates the findings by Li et al. [26], who also observed prolonged hospital stays in more severe COVID-19 cases.

Our study shows how the patients who experienced COVID-19 in the most severe form were those who had the most previous comorbidities. The data from studies such as Goyal et al.’s [27] revealed that people with pre-existing comorbidities, such as chronic lung diseases or diabetes, are more likely to develop a more severe form of COVID-19.

In relation to frailty, the CFS and FRAIL showed that frailty increased as the severity of the disease increased. Also using FRAIL, Ma et al. [28] found that frailty was associated with a higher risk of developing more severe disease in COVID-19 patients. This has clinical utility. A prior assessment of frailty can help doctors provide an early warning of a high risk of severe COVID-19 pneumonia. Furthermore, the systematic review and meta-analysis by Yang et al. [29] support these results. They found that frailty was significantly associated with greater COVID-19 severity, mortality, ICU admission, the application of invasive mechanical ventilation, and prolonged hospital stay.

Finally, we found that as patients transitioned from a lower severity group to a higher severity group, they experienced more dysphagia. Several studies [30] affirm that patients who spend the duration of the disease hospitalised, intubated, and with mechanical ventilation have a greater risk of dysphagia upon hospital discharge.

### 4.2. Discussion: Symptoms

As COVID-19 severity increased, patients experienced more fatigue, dyspnoea, pain, and poorer overall health. Though specific effects of severity on these symptoms have not been studied, research has explored the relationship between severity and other persistent symptoms. Xie et al. [31] found that the burden of post-COVID-19 symptoms increased with acute infection severity, and Kamal et al. [32] linked severe cases to more pronounced symptoms like strokes and kidney failure.

Although none of the groups exceeded the established cut-off point of the HADS for determining the presence of anxiety and/or depression post COVID-19, the milder cases showed a tendency to have less anxiety and depression than the more severe cases. These findings are consistent with studies suggesting that the severity of illness may influence the prevalence of post-COVID psychiatric disorders [33]. For example, Mazza et al. [34] identified significantly higher levels of anxiety and depression in patients with more severe acute infection. Similarly, Yıldızeli et al. [35] found that a higher severity of COVID-19 correlates positively with anxiety and depression.

### 4.3. Discussion: Physical Activity and Functionality

The IPAQ indicated that physical activity levels were highest in healthy individuals, followed by mild, severe, moderate, and critical COVID-19 patients. This variability could be due to subjective interpretations of “intense” or “moderate” physical activity based on individual fitness levels. This finding aligns with Huang et al. [36], who reported decreased physical activity in 61.4% of hospitalised COVID-19 patients, likely exacerbated by prolonged immobilisation during hospital stays.

Our study also revealed worse functionality, measured by the PSFS, among patients with more severe COVID-19. COVID-19 significantly impacts the ability to perform daily activities and maintain an active lifestyle, as supported by findings from Leite et al. [37] who concluded that hospitalisation predicts poorer functional outcomes post COVID-19 compared to non-hospitalised cases. Additionally, assessments using SPPB, the Arm Curl test, and the 2MST showed declining functionality with increasing COVID-19 severity. While specific studies on functionality relative to COVID-19 severity are limited, general research, such as that by De Oliveira et al. [38], underscores the higher prevalence of impaired functionality among COVID-19 survivors, emphasising the importance of multidisciplinary rehabilitation.

### 4.4. Limitations and Strengths

Limitations: The small sample size across different groups potentially compromised statistical precision and result validity. However, participant distribution across severity groups aligns with the WHO data on COVID-19 severity: “The majority of people with COVID-19 have mild (40%) or moderate (40%) disease, with approximately 15% requiring oxygen therapy for severe disease, and 5% critically ill with complications” [2]. There is a possible recall bias. Although specific questions were designed as much as possible to minimise interpretations or generalisations, and “double-check” questions were used, the complete elimination of this bias cannot be guaranteed. Furthermore, the administration of questionnaires online and data collection via video calls may have favoured participants with higher digital literacy and better access to technology, potentially limiting the representativeness of some populations. There was heterogeneity among groups, primarily due to older age and more comorbidities in severe cases [27]. Finally, it is impossible to completely rule out previous asymptomatic or undiagnosed COVID-19 infections in healthy participants, as no specific serological tests were performed to confirm the absence of previous exposure to the virus.

Strengths: This study is the first to evaluate COVID-19 impact across severity groups. Evaluators were rigorously trained, adhering to a strict protocol. The STROBE guideline was followed. This study serves as a solid foundation for future research on post-COVID-19 symptom treatments tailored to disease severity.

### 4.5. Relevance for Clinical Practise

The findings of our study highlight the different impact that COVID-19 has had on patients depending on the severity of disease. It is essential that this be considered for future studies when developing rehabilitation protocols for post-COVID-19 symptoms. These treatments, in addition to being adapted to the severity with which the disease has occurred, should present a multidisciplinary approach. This is indispensable to be able to act on the entire broad spectrum of persistent symptoms left by COVID-19.

## 5. Conclusions

The impact of COVID-19 on surviving patients varies significantly with the severity of the disease. The results show that the hospitalisation time, age, and comorbidities of the patients are greater in those with a greater severity of the disease. Patients with more severe COVID-19 also experience greater frailty, dysphagia, fatigue, dyspnoea, and pain. Additionally, those with severe cases have poorer overall health, reduced physical activity, and diminished functionality. No evidence of post-COVID-19 anxiety or depression is found in the sample, even considering the timeframe between the negative test and the assessment. However, the milder cases show a tendency to have less anxiety and depression more than the more severe cases.

Given the high number of patients experiencing post-COVID-19 symptoms, rehabilitation protocols are being developed. The findings of this study are crucial for tailoring these rehabilitation programmes. Treatment should be personalised based on the severity of COVID-19 and the specific described characteristics of each patient.

It is recommended that future studies consider larger and more representative samples, including, for example, age- and sex-stratified analyses, in order to explore in more detail how COVID-19 severity affects different subpopulations. In addition, the integration of a face-to-face clinical test would allow for the greater accuracy of results and more comprehensive analysis. These efforts would contribute significantly to complement the findings of our study.

## Figures and Tables

**Table 1:** Descriptive characteristics of participants.

VARIABLE	HEALTHY (*n* = 42) Mean ± SD	MILD (*n* = 143) Mean ± SD	MODERATE (*n* = 49) Mean ± SD	SEVERE (*n* = 52) Mean ± SD	CRITICAL (*n* = 18) Mean ± SD	F VALUE
Age (years)	32.48 ± 15.00	39.97 ± 15.81	40.88 ± 14.77	61.46 ± 12.75	66.17 ± 9.56	37.998 ^b,c,d,e,f,i,j^
Gender male *n* (%)	19 (45.20)	40 (28.00)	16 (32.70)	33 (63.50)	9 (50.00)	22.899 **
BMI (Kg/m^2^)	24.10 ± 3.67	24.51 ± 4.21	25.91 ± 4.91	29.07 ± 5.12	28.97 ± 5.10	13.605 ^b,c,e,i,j^
Days between negative test and assessment date	-	204.13 ± 153.29	237.22 ± 220.86	513.29 ± 409.98	495.33 ± 350.92	-
Days of hospitalisation	-	-	-	11.40 ± 6.42	38.89 ± 37.04	-
Days in ICU	-	-	-	-	23.94 ± 26.18	-
Comorbidities (Charlson Comorbidity Index)	0.38 ± 0.66	1.01 ± 1.49	1.12 ± 1.74	2.90 ± 2.22	3.56 ± 2.23	24.820 ^b,c,e,f,i,j^
Frailty						
CFS (score)	1.67 ± 0.79	2.30 ± 0.89	2.78 ± 1.07	3.67 ± 1.31	3.56 ± 1.62	29.367 ^b,c,d,e,g,i,j^
FRAIL (score)	0.19 ± 0.46	0.43 ± 0.74	0.88 ± 0.86	1.44 ± 1.32	1.67 ± 1.50	20.517 ^a,b,c,e,f,g,i,j^
Dysphagia (EAT-10)	0.17 ± 0.54	0.46 ± 1.68	1.84 ± 4.62	2.23 ± 5.23	4.67 ± 9.99	6.840 ^c,j^

** *p* < 0.001; ^a^ significant difference between mild and moderate group; ^b^ significant difference between mild and severe group; ^c^ significant difference between mild and critical group; ^d^ significant difference between mild and healthy group; ^e^ significant difference between moderate and severe group; ^f^ significant difference between moderate and critical group; ^g^ significant difference between moderate and healthy group; ^i^ significant difference between severe and healthy group; ^j^ significant difference between critical and healthy group; BMI: Body Mass Index; CFS: Clinical Frailty Scale; EAT-10: Eating Assessment Tool-10; FRAIL: FRAIL frailty scale; ICU: intensive care unit; SD: standard deviation.

**Table 2:** Symptoms according to severity.

VARIABLE	HEALTHY (*n* = 42) Mean ± SD	MILD (*n* = 143) Mean ± SD	MODERATE (*n* = 49) Mean ± SD	SEVERE (*n* = 52) Mean ± SD	CRITICAL (*n* = 18) Mean ± SD	F VALUE
Fatigue						
Borg scale						
Resting	0.21 ± 0.51	0.67 ± 1.57	2.28 ± 2.97	1.29 ± 2.55	1.72 ± 2.88	8.015 ^a,g^
In light exercise	0.87 ± 0.96	1.56 ± 2.19	3.81 ± 3.21	4.04 ± 3.60	5.61 ± 3.07	22.735 ^a,b,c,g,i,j^
In moderate exercise	2.39 ± 1.90	3.04 ± 2.71	5.33 ± 3.04	6.41 ± 3.53	7.56 ± 2.64	26.646 ^a,b,c,f,g,i,j^
In intense exercise	4.42 ± 2.38	4.75 ± 2.90	6.57 ± 2.90	7.87 ± 3.31	8.81 ± 2.48	19.518 ^a,b,c,g,i,j^
FIS						
Physical effort	7.36 ± 4.71	10.80 ± 8.46	20.53 ± 9.50	21.52 ± 11.96	21.72 ± 7.86	29.550 ^a,b,c,g,i,j^
Cognitive effort	7.67 ± 6.54	10.53 ± 8.12	16.04 ± 8.98	15.56 ± 12.65	13.72 ± 11.72	7.646 ^a,b,g,i^
Psychosocial effort	1.31 ± 1.73	1.74 ± 1.90	3.69 ± 2.48	4.23 ± 3.38	3.78 ± 2.53	18.273 ^a,b,c,g,i,j^
Total score	16.33 ± 10.60	23.07 ± 16.47	40.27 ± 18.15	41.31 ± 24.84	39.22 ± 20.04	21.332 ^a,b,c,g,i,j^
FSS	24.79 ± 9.38	26.44 ± 14.63	41.82 ± 15.80	41.88 ± 20.14	46.50 ± 16.17	21.148 ^a,b,c,g,i,j^
Dyspnoea (Borg scale)						
Resting	0.12 ± 0.38	0.37 ± 1.06	1.52 ± 2.10	1.17 ± 2.34	1.17 ± 2.20	7.932 ^a,b,g,i^
In light exercise	0.48 ± 0.68	1.03 ± 1.80	2.88 ± 2.65	3.70 ± 3.78	3.44 ± 3.54	19.575 ^a,b,c,g,i,j^
In moderate exercise	1.55 ± 1.47	2.30 ± 2.38	4.89 ± 2.97	5.90 ± 3.90	6.22 ± 3.51	29.120 ^a,b,c,g,i,j^
In intense exercise	3.88 ± 2.52	4.17 ± 2.88	6.47 ± 2.80	7.28 ± 3.80	7.56 ± 3.28	17.370 ^a,b,c,g,i,j^
Pain (VAS)	1.33 ± 2.21	1.51 ± 2.36	2.78 ± 3.37	3.04 ± 3.33	3.67 ± 3.65	5.997 ^b,c,i,j^
Anxiety and depression (HADS)						
Anxiety	3.62 ± 1.99	5.52 ± 4.01	7.84 ± 5.34	7.02 ± 5.50	5.44 ± 6.68	6.019 ^a,g,i^
Depression	1.45 ± 1.76	2.52 ± 2.77	4.82 ± 4.29	5.06 ± 4.74	5.67 ± 6.63	11.270 ^a,b,c,g,i,j^
Total score	5.07 ± 3.02	8.04 ± 6.11	12.65 ± 9.10	12.08 ± 9.61	11.11 ± 12.53	8.718 ^a,b,g,i,j^
Quality of life (EuroQol-5D)						
Mobility	1.02 ± 0.15	1.06 ± 0.24	1.08 ± 0.28	1.27 ± 0.53	1.39 ± 0.50	7.994 ^b,c,e,f,i,j^
Personal care	1.02 ± 0.15	1.03 ± 0.17	1.06 ± 0.24	1.23 ± 0.58	1.11 ± 0.32	4.814 ^b,e,i^
Daily activities	1.02 ± 0.15	1.07 ± 0.26	1.18 ± 0.39	1.40 ± 0.66	1.44 ± 0.51	10.921 ^b,c,e,i,j^
Pain/discomfort	1.40 ± 0.59	1.40 ± 0.58	1.63 ± 0.76	1.75 ± 0.81	1.94 ± 0.80	5.143 ^b,c,j^
Anxiety/depression	1.17 ± 0.38	1.31 ± 0.52	1.55 ± 0.71	1.52 ± 0.73	1.56 ± 0.78	3.993 ^g,i^
Health status (VAS)	84.40 ± 10.66	78.41 ± 16.10	63.63 ± 21.41	61.90 ± 23.94	60.00 ± 25.50	16.855 ^a,b,c,g,i,j^

^a^ Significant difference between mild and moderate group; ^b^ significant difference between mild and severe group; ^c^ significant difference between mild and critical group; ^e^ significant difference between moderate and severe group; ^f^ significant difference between moderate and critical group; ^g^ significant difference between moderate and healthy group; ^i^ significant difference between severe and healthy group; ^j^ significant difference between critical and healthy group; EuroQol-5D: European Quality of Life-5 Dimensions; FIS: Fatigue Impact Scale; FSS: Fatigue Severity Scale; HADS: Hospital Anxiety and Depression Scale; SD: standard deviation; VAS: Visual Analogue Scale.

**Table 3:** Physical activity and functionality.

VARIABLE	HEALTHY (*n* = 42) Mean ± SD	MILD (*n* = 143) Mean ± SD	MODERATE (*n* = 49) Mean ± SD	SEVERE (*n* = 52) Mean ± SD	CRITICAL (*n* = 18) Mean ± SD	F VALUE
PA (IPAQ) (METs/min/week)						
Weekly metabolic rate in intense PA	1840.00 ± 2016.13	1282.24 ± 2391.04	759.18 ± 1382.47	1080.00 ± 3283.67	80.00 ± 246.958	2.339
Weekly metabolic rate in moderate PA	871.43 ± 1216.87	702.38 ± 1590.07	663.67 ± 1213.94	480.00 ± 1103.15	353.33 ± 821.62	0.731
Weekly metabolic rate in light PA (walks)	711.29 ± 1527.19	1404.69 ± 1650.62	1078.90 ± 1095.03	1043.31 ± 1408.07	1578.50 ± 1671.65	1.664
Global weekly metabolic rate	4422.71 ± 3323.81	3389.31 ± 4037.87	2501.76 ± 2627.65	2603.31 ± 4143.81	2011.83 ± 1927.93	2.508
Functionality						
PSFS	4.38 ± 1.71	4.69 ± 1.93	4.13 ± 2.09	3.47 ± 2.18	3.19 ± 1.91	5.211 ^b,c^
Short Physical Performance Battery						
Balance test	3.98 ± 0.15	3.95 ± 0.33	3.83 ± 0.70	3.78 ± 0.66	3.71 ± 0.83	2.054
Walking speed	3.76 ± 0.53	3.35 ± 0.83	3.39 ± 0.80	3.10 ± 0.94	3.00 ± 0.88	4.318 ^d,i,j^
Strength	3.26 ± 0.91	2.78 ± 1.11	2.68 ± 1.23	1.81 ± 0.97	1.83 ± 0.84	11.226 ^b,c,e,i,j^
Total score	11.00 ± 1.19	9.89 ± 1.82	9.69 ± 2.24	8.48 ± 1.85	8.29 ± 1.64	12.848 ^b,c,d,e,g,i,j^
Arm Curl test						
Right arm	19.83 ± 4.85	17.86 ± 5.73	17.15 ± 6.70	14.41 ± 6.09	12.71 ± 5.24	6.838 ^b,c,i,j^
Left arm	20.43 ± 4.86	18.48 ± 5.72	18.10 ± 7.49	15.42 ± 5.98	14.00 ± 5.40	5.418 ^i,j^
2 min step test	108.69 ± 18.54	102.47 ± 24.20	99.36 ± 30.26	86.52 ± 19.60	82.44 ± 18.45	5.301 ^b,i,j^

^b^ Significant difference between mild and severe group; ^c^ significant difference between mild and critical group; ^d^ significant difference between mild and healthy group; ^e^ significant difference between moderate and severe group; ^g^ significant difference between moderate and healthy group; ^i^ significant difference between severe and healthy group; ^j^ significant difference between critical and healthy group; IPAQ: International Physical Activity Questionnaire; PA: physical activity; PSFS: Patient-Specific Functional Scale; SD: Standard deviation.

## Data Availability

The raw data supporting the conclusions of this article will be made available by the authors on request.

## Appendix A. STROBE Statement—Checklist of Items That Should Be Included in Reports of Observational Studies

	Item Nº	Recommendation	Location
Title and abstract	1	(a) Indicate the study’s design with a commonly used term in the title or the abstract	Pg. 1
		(b) Provide in the abstract an informative and balanced summary of what was done and what was found	Pg. 1
Introduction			
Background/rationale	2	Explain the scientific background and rationale for the investigation being reported	Pgs. 2, 3
Objectives	3	State specific objectives, including any prespecified hypotheses	Pg. 3
Methods			
Study design	4	Present key elements of study design early in the paper	Pg. 3
Setting	5	Describe the setting, locations, and relevant dates, including periods of recruitment, exposure, follow-up, and data collection	Pg. 3
Participants	6	(a) Cohort study—Give the eligibility criteria, and the sources and methods of selection of participants. Describe methods of follow-up Case–control study—Give the eligibility criteria, and the sources and methods of case ascertainment and control selection. Give the rationale for the choice of cases and controls Cross-sectional study—Give the eligibility criteria, and the sources and methods of selection of participants	Pg. 3
		(b) Cohort study—For matched studies, give matching criteria and number of exposed and unexposed Case–control study—For matched studies, give matching criteria and the number of controls per case	-
Variables	7	Clearly define all outcomes, exposures, predictors, potential confounders, and effect modifiers. Give diagnostic criteria, if applicable	Pg. 4
Data sources/measurement	8	For each variable of interest, give sources of data and details of methods of assessment (measurement). Describe comparability of assessment methods if there is more than one group	Pg. 4
Bias	9	Describe any efforts to address potential sources of bias	Pg. 3
Study size	10	Explain how the study size was arrived at	Pgs. 3, 4
Quantitative variables	11	Explain how quantitative variables were handled in the analyses. If applicable, describe which groupings were chosen and why	Pgs. 4, 5
Statistical methods	12	(a) Describe all statistical methods, including those used to control for confounding	Pgs. 4, 5
		(b) Describe any methods used to examine subgroups and interactions	-
		(c) Explain how missing data were addressed	Pgs. 4, 5
		(d) Cohort study—If applicable, explain how loss to follow-up was addressed Case–control study—If applicable, explain how matching of cases and controls was addressed Cross-sectional study—If applicable, describe analytical methods taking account of sampling strategy	-
		(e) Describe any sensitivity analyses	Pgs. 4, 5
Results			
Participants	13	(a) Report numbers of individuals at each stage of study—e.g., numbers potentially eligible, examined for eligibility, confirmed eligible, included in the study, completing follow-up, and analysed	Pgs. 3, 5 and Figure 1
		(b) Give reasons for non-participation at each stage	Figure 1
		(c) Consider use of a flow diagram	Figure 1
Descriptive data	14	(a) Give characteristics of study participants (e.g., demographic, clinical, social) and information on exposures and potential confounders	Pg. 5 and Table 1
		(b) Indicate number of participants with missing data for each variable of interest	Table 1, Table 2 and Table 3
		(c) Cohort study—Summarise follow-up time (e.g., average and total amount)	-
Outcome data	15	Cohort study—Report numbers of outcome events or summary measures over time	-
		Case–control study—Report numbers in each exposure category, or summary measures of exposure	-
		Cross-sectional study—Report numbers of outcome events or summary measures	Table 1, Table 2 and Table 3
Main results	16	(a) Give unadjusted estimates and, if applicable, confounder-adjusted estimates and their precision (e.g., 95% confidence interval). Make clear which confounders were adjusted for and why they were included	Table 1, Table 2 and Table 3
		(b) Report category boundaries when continuous variables were categorized	Pgs. 5–11
		(c) If relevant, consider translating estimates of relative risk into absolute risk for a meaningful time period	-
Other analyses	17	Report other analyses done—e.g., analyses of subgroups and interactions, and sensitivity analyses	-
Discussion			
Key results	18	Summarise key results with reference to study objectives	Pgs. 12, 13
Limitations	19	Discuss limitations of the study, taking into account sources of potential bias or imprecision. Discuss both direction and magnitude of any potential bias	Pg. 13
Interpretation	20	Give a cautious overall interpretation of results considering objectives, limitations, multiplicity of analyses, results from similar studies, and other relevant evidence	Pgs. 12, 13
Generalisability	21	Discuss the generalisability (external validity) of the study results	Pgs. 12, 13
Other information			
Funding	22	Give the source of funding and the role of the funders for the present study and, if applicable, for the original study on which the present article is based	Pg. 14
